# A daily regimen of a ceramide‐dominant moisturizing cream and cleanser restores the skin permeability barrier in adults with moderate eczema: A randomized trial

**DOI:** 10.1111/dth.14970

**Published:** 2021-05-24

**Authors:** Fabrizio Spada, Ian P. Harrison, Tanya M. Barnes, Kerryn A. Greive, Daisy Daniels, Joshua P. Townley, Niyaz Mostafa, Andrew T. Fong, Philip L. Tong, Stephen Shumack

**Affiliations:** ^1^ Department of Scientific Affairs Ego Pharmaceuticals Melbourne Victoria Australia; ^2^ St George Dermatology and Skin Cancer Centre Sydney New South Wales Australia; ^3^ Faculty of Medicine University of New South Wales Sydney New South Wales Australia; ^4^ The Children's Hospital at Westmead Westmead, Sydney New South Wales Australia; ^5^ Department of Dermatology St Vincent's Hospital Sydney New South Wales Australia; ^6^ The Skin Hospital Sydney New South Wales Australia; ^7^ Department of Dermatology Royal North Shore Hospital Sydney New South Wales Australia

**Keywords:** atopic dermatitis, dermatology life quality index, eczema area severity index, hydration, transepidermal water loss

## Abstract

The dysfunctional skin barrier in eczema patients may be attributed to decreased levels of ceramides in the stratum corneum. The aim of this study was to determine whether a two‐part system consisting of a ceramide‐dominant physiological lipid‐based moisturizing cream and cleanser could ameliorate the signs and symptoms of moderate eczema in adults over 28 days compared to placebo. Assessments were conducted at baseline and every 7 days thereafter. Eczema area severity index score decreased significantly across all time points in both groups compared to baseline (*P* < .0001), however, this decrease was not significant between groups at day 28 (*P* = .7804). In contrast, transepidermal water loss and skin hydration significantly improved over time in the active group, while it either stayed the same or worsened in the placebo group (*P* = .0342 and *P* < .0001, respectively). There was no difference in the use of mometasone furoate as rescue medication over time between groups (*P* = .1579). Dermatology life quality index scores improved significantly in both groups (*P* < .0001), with no difference between groups (*P* = .5256). However, patient satisfaction was greater in the active compared to the placebo group for several parameters including relief of itch, dry skin, skin softness and smoothness (all *P* < .05). No patients withdrew from the study due to adverse events (AEs) and there were no serious AEs. The ceramide‐dominant moisturizing cream and cleanser safely restores skin permeability and improves the signs and symptoms of eczema in adults.

## 1 INTRODUCTION

Atopic dermatitis (AD), also known as eczema, is a chronic, relapsing, inflammatory skin disease characterized by a broad spectrum of clinical manifestations such as erythema, xerosis, intense pruritus or itch, and a dysfunctional epidermal skin barrier.^1^ The compromised skin barrier is mainly attributable to significantly decreased levels of ceramides in the stratum corneum (SC) in lesional and non‐lesional skin.^2^, ^3^ Ceramides act as water modulators and an integral part of the skins permeability barrier by forming multi‐layered lamellar structures with cholesterol and free fatty acids between cells of the SC.^4^ The abnormal barrier function in eczema results in increased transepidermal water loss (TEWL) leading to xerosis, and predisposes the skin to inflammatory processes evoked by irritants and allergens.^5^, ^6^ In addition to ceramide deficiency, changes in ceramide profiles including ceramide chain length have been linked with the impaired SC barrier function in eczema.^7^, ^8^

Eczema treatments have traditionally included topical corticosteroids and immunomodulators that do not target the underlying structural barrier abnormalities, and have clinically well‐recognized undesirable side effects.^9^ More recently it has been established that a crucial eczema management tool, including between episodes of flare ups, is the frequent use of an appropriate moisturizer.^10^ However, most conventional moisturizers do not address the underlying lipid deficiency in eczematous skin.^11^ Conventional moisturizers form a more superficial occlusive barrier on the skin whereas physiologic lipids, including ceramides, permeate the SC and are synthesized in the keratinocytes, processed in lamellar bodies, and secreted back into the SC to become a part of the dermal matrix.^12^ As such, and coupled with an improved understanding of the etiology of eczema, new pharmacological approaches should focus on correcting the epidermal barrier dysfunction through the inclusion of specific SC lipids at the appropriate concentration in moisturizers.^13^, ^14^

The objective of this randomized, double‐blind, placebo‐controlled, single centre, comparative trial was to determine whether a two‐part system consisting of a ceramide‐dominant physiological lipid‐based moisturizing cream and cleanser, could safely ameliorate the signs and symptoms of moderate eczema in adult patients compared to placebo over 28 days. Efficacy was determined through the evaluation of eczema area severity index (EASI), TEWL, and skin hydration. In addition, patients completed the dermatology life quality index (DLQI) survey as well as a patient satisfaction survey. Safety of the study products was also closely monitored.

## 2 METHODS

The study was entered in the Australian New Zealand Clinical Trial Registry on July 28, 2015 (registration number: ACTRN12615000782538). Ethics approval was obtained from Bellberry Limited (Eastwood, South Australia, Australia), which operates in accordance with the National Health and Medical Research Council of Australia's National Statement on Ethical Conduct in Human Research, the World Medical Association Declaration of Helsinki and the International Conference on Harmonization Good Clinical Practice guidelines.

### 2.1 Study products

QV intensive with ceramides light moisturizing cream (ceramide cream) and QV intensive with ceramides hydrating body wash (ceramide cleanser) were obtained from Ego Pharmaceuticals Pty. Ltd. (Braeside, Victoria, Australia). Placebo cream and placebo cleanser were formulated without the skin active ingredients (Table 1).

**TABLE 1:** List of ingredients in the study products

	Ceramide cream	Placebo cream	Ceramide cleanser	Placebo cleanser
Base	water	water	water	water
Humectant	glycerin		glycerin	
	sodium PCA^a^		sodium PCA^a^	
Occludent	dimethicone		petrolatum	
	petrolatum			
Emollient	paraffinium liquidum		1,2‐hexanediol	
	1,2‐hexanediol		caprylyl glycol	
	caprylyl glycol			
Ceramide promoter	niacinamide		niacinamide	
	lactic acid^a^		lactic acid^a^	
Lipid	ceramide NP		ceramide NP	
	ceramide EOP		ceramide EOP	
	cholesterol		cholesterol	
	*Carthamus tinctorius* (safflower) seed oil		*Carthamus Tinctorius* (safflower) seed oil	
Other	cetearyl alcohol	cetearyl alcohol	lauryl betaine	lauryl betaine
	ceteareth‐20	ceteareth‐20	sodium cocoyl isethionate	sodium cocoyl isethionate
	glyceryl stearate SE	glyceryl stearate SE	sodium lauroyl sarcosinate	sodium lauroyl sarcosinate
	laureth‐3	laureth‐3	sodium polyacrylate	sodium polyacrylate
	sodium hydroxide	methylparaben	xanthan gum	styrene/acrylates copolymer
	stearic acid	propylparaben		xanthan gum
	xanthan gum	stearic acid		
		xanthan gum		

^a^ also a component of the natural moisturizing factor (NMF).

### 2.2 Patient population

A total of 100 patients were recruited from the outpatient clinic at St George Dermatology and Skin Cancer Centre (Kogarah, New South Wales, Australia) between September 2015 and October 2019. Inclusion criteria were: (a) males or females aged over 18 years, (b) clinically diagnosed eczema for at least 1 year according to the criteria of Hanifin & Raijka,^15^ with moderate severity (score of 10‐20) as evaluated by EASI,^16^ (c) free of any dermatological or systemic disorder which could interfere with results and (d) free of any acute or chronic disease that may interfere with or increase the risk of trial participation. The exclusion criteria were: (a) history of allergies or adverse reactions to moisturizers or components of the specific products being tested, (b) use of any medication (topical or systemic) that may mask or interfere with results, such as calcineurin inhibitors or corticosteroids, (c) excessive hair on test sites, (d) history of chronic allergies and (e) pregnant or nursing females.

Patients were instructed not to use their usual moisturizers, cleansers or topical medications for 1 week prior to participation (wash‐out) or during the study period. All patients gave their written informed consent prior to participation.

### 2.3 Study design

**FIGURE 1:**
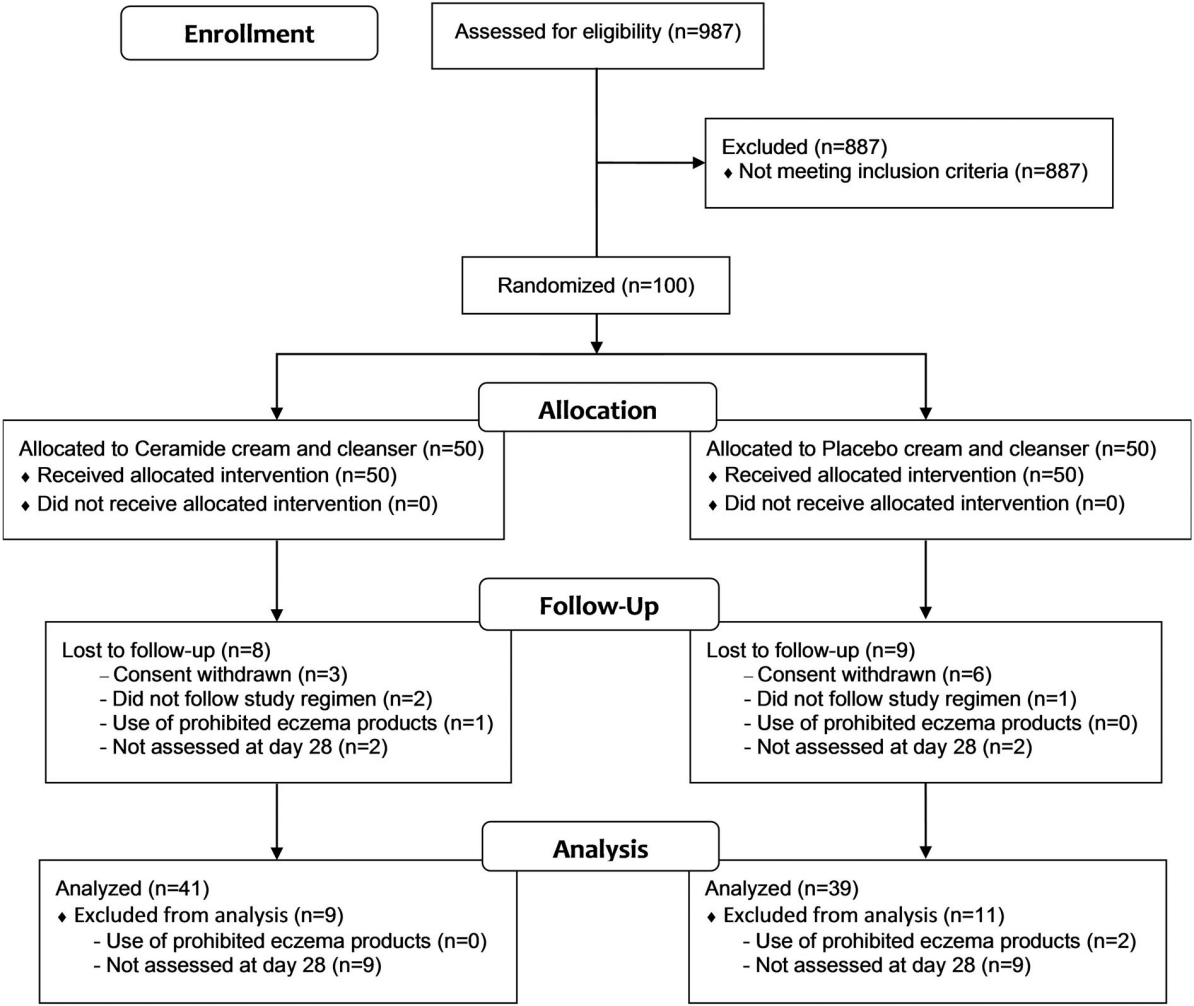
Flow chart illustrating the progress of participants through the study

Patients meeting the inclusion criteria were randomly assigned to receive either the ceramide cream and ceramide cleanser or placebo cream and placebo cleanser according to a randomization schedule generated using SAS statistical software (SAS Institute Inc.) blocked in groups of six (Figure 1). The study products were filled into identical 350 mL pump pack containers and each assigned a randomization code to conceal their identity from participants and the physician allocating treatments and assessing outcomes. Data analysts were also blinded to the identification of each study group until the final efficacy analyses were complete.

Patients were instructed to massage the cleanser into wet skin across their whole body once daily, rinse with water and pat dry, for 28 days. Patients were also required to apply the cream liberally to their whole body, in particular eczema‐prone areas, twice daily, morning and night for 28 days. One application of cream was required after daily body cleansing. In addition, patients were supplied with mometasone furoate 0.1% w/w (Zatamil Hydrogel; Ego Pharmaceuticals Pty. Ltd., Braeside, Victoria, Australia) to be used as rescue medication for the duration of the study in place of their usual topical corticosteroids or calcineurin inhibitors.

### 2.4 Efficacy and safety assessments

The primary efficacy outcome was comparison of the treatments effects on symptom severity as assessed by EASI at day 28 compared to baseline. The EASI combines the severity of four signs of eczema (redness, thickness/swelling, itching, lichenification) and the extent of skin involvement at four body regions (head/neck, upper limbs, trunk and lower limbs), with the composite score ranging from 0 to 72.

Secondary efficacy outcomes were the comparison of the treatments based on EASI at days 7, 14, and 21 compared to baseline, as well as the change in TEWL, skin hydration and the amount of mometasone furoate used as rescue medication at days 7, 14, 21, and 28 compared to baseline. Furthermore, the comparison of the treatments based on the DLQI survey as well as a patient satisfaction survey at days 14 and 28 compared to baseline were also determined. The DLQI is a 10 item questionnaire focusing on how eczema affects overall life quality rated using a four point scale from “not at all” to “very much”.^17^ The patient satisfaction survey is a 9 item questionnaire which was designed to focus on eczema symptoms and satisfaction with the treatments, rated on the same scale.

Prior to measurements of skin biophysical properties, participants were required to equilibrate in a closed environment with a constant temperature (20°C ± 2°C) and humidity (45 to 55% RH). Measurement of TEWL was performed using a tewameter (Model TM 210, Courage and Khazaka, Germany),^18^ while skin hydration was measured using a corneometer (Model CM 825, Courage and Khazaka, Germany)^19^ at five different points on the skin of the forearm and the mean value recorded.

All adverse events (AEs), including serious AEs, were recorded and carefully monitored until they were resolved or the patient's participation in the study ended. The site physician assessed the seriousness of any AE and the relationship of the AE to the study products.

### 2.5 Statistical analysis

SAS Software, Version 9.4 was used to perform the statistical analysis. A power analysis was used to determine the number of participants required for the study. Assuming an alpha (α) of 0.05, power (1‐β) of 0.8, a difference between group means of 12% and a SD of 20, approximately 42 participants per treatment group was calculated to be required.

Student's *t* test were used to test for statistically significant differences (*P* < .05) between time points in EASI score, TEWL, skin hydration, DLQI and quantity of corticosteroid used in the active vs placebo group. Repeated measures analysis of covariance in a mixed models framework with baseline value as a covariate was used to perform trend analyses and further comparisons. Responses on the patient satisfaction survey were analyzed using cumulative logistic regression.

## 3 RESULTS

### 3.1 Study population

The intention‐to‐treat (ITT) population included all participants who were randomized and received at least one dose of the study products. The demographics and baseline characteristics of the ITT population are described in Table 2 and consisted of 100 patients (53 female, 47 male; aged 18‐73 years; mean age 30.9 years) who received either ceramide cream and cleanser (n = 50) or placebo cream and cleanser (n = 50). Of these, 83 patients completed the study (n = 42 and n = 41, respectively). Early withdrawal from the study was due to consent being withdrawn (n = 3 and n = 6, respectively), not following the study regimen (n = 2 and n = 1, respectively), use of prohibited eczema products (n = 1 and n = 0, respectively) and not assessed on day 28 (n = 2 and n = 2, respectively) (Figure 1). The per‐protocol (PP) population included 41 and 39 patients in the active and placebo groups, respectively. Protocol deviations leading to exclusion from the PP population included use of prohibited eczema products (n = 0 and n = 2, respectively) and not assessed on day 28 (n = 9 and n = 9, respectively) (Figure 1), resulting in the ITT and PP populations differing by just n = 1 and n = 2, respectively. Therefore, only results for the ITT population are presented. One participant found that she was pregnant during the course of the study, however, the pregnancy was deemed unlikely to significantly impact the efficacy results so the participant's data was included in the analysis.

**TABLE 2:** Patient demographics and baseline characteristics by treatment group (ITT population)

	Ceramide cream and cleanser (n = 50)	Placebo cream and cleanser (n = 50)	Total (n = 100)
Sex			
Male	25 (50%)	22 (56%)	53 (53%)
Female	25 (50%)	28 (44%)	47 (47%)
Age (y)			
Mean ± SD (Range)	29.6 ± 10.6 (18‐63)	32.2 ± 14.5 (18–73)	30.9 ± 12.7 (18‐73)
Race			
Asian	27 (54%)	22 (44%)	49 (49%)
Black	0 (0%)	1 (2%)	1 (1%)
Caucasian	13 (26%)	17 (34%)	30 (30%)
Hispanic	0 (0%)	1 (2%)	1 (1%)
Other	8 (16%)	5 (10%)	13 (13%)
Missing	2 (4%)	4 (8%)	6 (6%)
Skin allergies/sensitivities			
Perfumes/Fragrance	16 (32%)	15 (30%)	31 (31%)
Soaps/Laundry detergents	20 (40%)	21 (42%)	41 (41%)
Cosmetics	12 (24%)	15 (30%)	27 (27%)
Antiperspirants/Deodorants	10 (20%)	6 (12%)	16 (16%)
Foods	18 (36%)	18 (36%)	36 (36%)
Medicines	3 (6%)	4 (8%)	7 (7%)
Adhesives (Band‐aids)	2 (4%)	3 (6%)	5 (5%)
Suntan products/Sunscreen	2 (4%)	11 (22%)	13 (13%)
Aspirin	0 (0%)	2 (4%)	2 (2%)
Anything else	7 (14%)	9 (18%)	16 (16%)
Medical diagnoses			
Eczema	50 (100%)	50 (100%)	100 (100%)
Diabetes	2 (4%)	1 (2%)	3 (3%)
Asthma	18 (36%)	21 (42%)	39 (39%)
Hayfever/Allergies	31 (62%)	33 (66%)	64 (64%)
Psoriasis	1 (2%)	3 (6%)	4 (4%)
Dandruff	12 (24%)	12 (24%)	24 (24%)
Cancer	0 (0%)	1 (2%)	1 (1%)
Arthritis	1 (2%)	0 (0%)	1 (1%)
Tinea pedis (Athletes foot)	2 (4%)	0 (0%)	2 (2%)
Heart trouble	1 (2%)	0 (0%)	1 (1%)
High blood pressure	5 (10%)	3 (6%)	8 (8%)
Anaphylactic reactions	3 (6%)	4 (8%)	7 (7%)
Epilepsy/Seizures	0 (0%)	2 (4%)	2 (2%)
Gastric ulcers	2 (4%)	1 (2%)	3 (3%)
Recurrent Headaches	5 (10%)	3 (6%)	8 (8%)
Other	0 (0%)	4 (8%)	4 (4%)
EASI score			
Mean ± SEM (Range)	14.70 ± 0.52 (10.0‐25.2)	14.28 ± 0.43 (10.0‐19.8)	14.49 ± 0.48 (10.0–25.2)
TEWL (g/hm^2^)			
Mean ± SEM (Range)	130.92 ± 7.14 (49.95‐250.88)	137.99 ± 9.99 (43.34‐307.79)	ND
Skin hydration			
Mean ± SEM (Range)	124.0 ± 8.94 (18‐303)	147.2 ± 10.6 (15‐328)	ND
DLQI score			
Mean ± SEM (Range)	12.8 ± 0.89 (1‐27)	11.7 ± 0.79 (2‐22)	ND

Abbreviations: ITT, intention‐to‐treat; ND, not determined.

Age and gender were approximately balanced between the two groups, and the majority of participants were either Asian or Caucasian. The most common skin allergies/sensitivities were to soaps, followed by food, perfumes, cosmetics, deodorants and sunscreen, which were well distributed between groups. Twenty‐six participants did not report any allergies/sensitivities at baseline. The most common non‐eczema conditions were hay fever/allergies, asthma and dandruff, which were also well distributed between groups.

### 3.2 Efficacy assessment

Baseline EASI scores were matched between groups (*P* > .05), however the placebo group had slightly less variance in scores overall due to two outliers (Table 2). For the primary efficacy outcome, both ceramide cream and cleanser (day 0:14.70 ± 0.52 vs day 28:8.25 ± 0.78, *P* < .0001) and placebo cream and cleanser (day 0:14.28 ± 0.43 vs day 28:7.84 ± 0.75, *P* < .0001) significantly decreased EASI score after 28 days, however, this change was not significantly different between groups (*P* = .7804).

**FIGURE 2:**
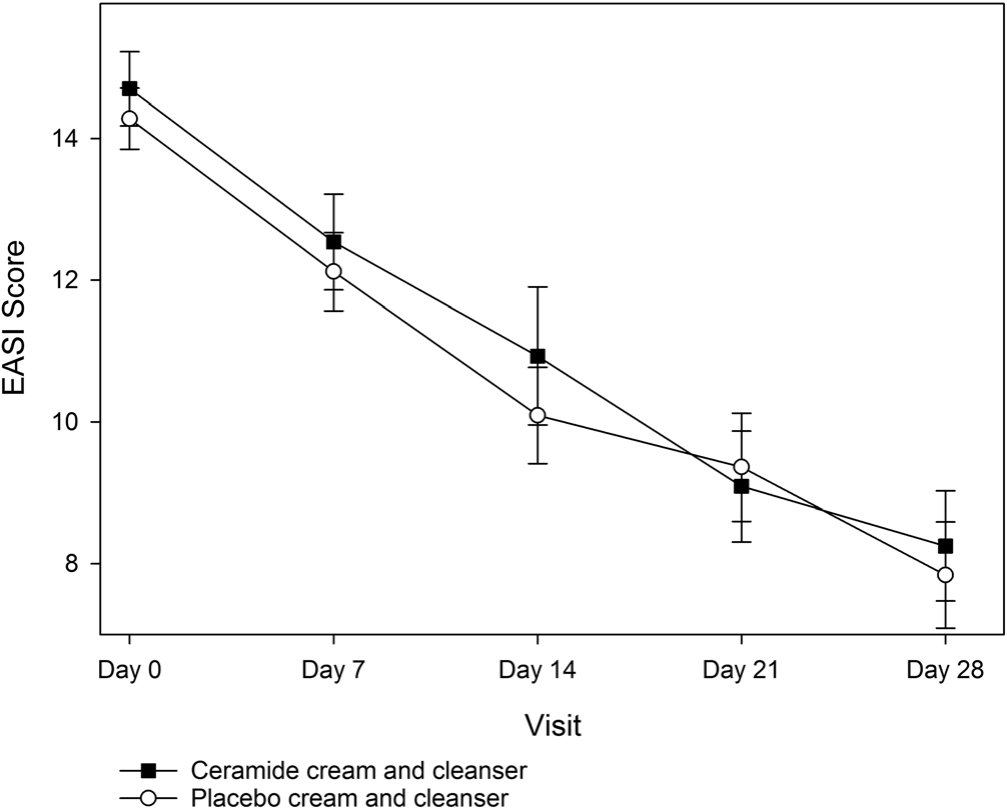
Eczema area severity index (EASI) score in the ceramide cream and cleanser and placebo cream and cleanser groups over the 28 day study period (ITT population)

For the secondary efficacy outcomes, EASI scores significantly improved across visits in both groups (*P* < .0001; Figure 2), however, there were no differences in the change in EASI score between the active and placebo groups at any time point (all *P* > .05).

**FIGURE 3:**
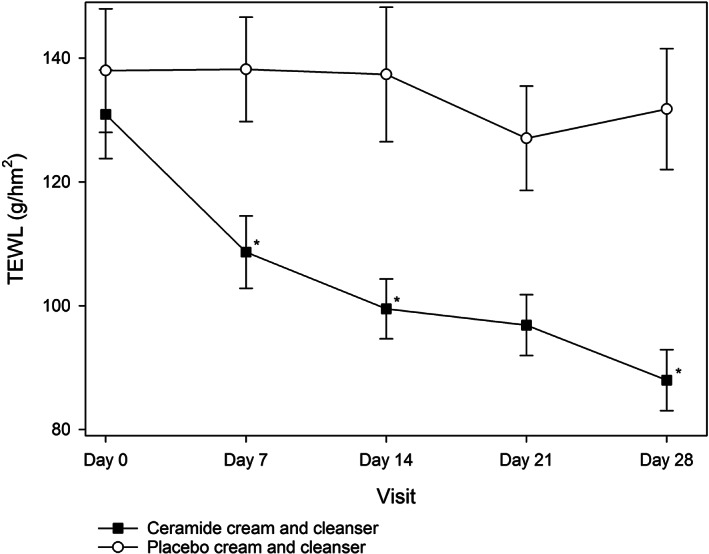
Transepidermal water loss (TEWL) in the ceramide cream and cleanser and placebo cream and cleanser groups over the 28 day study period (ITT population). *P < .05 vs placebo

**FIGURE 4:**
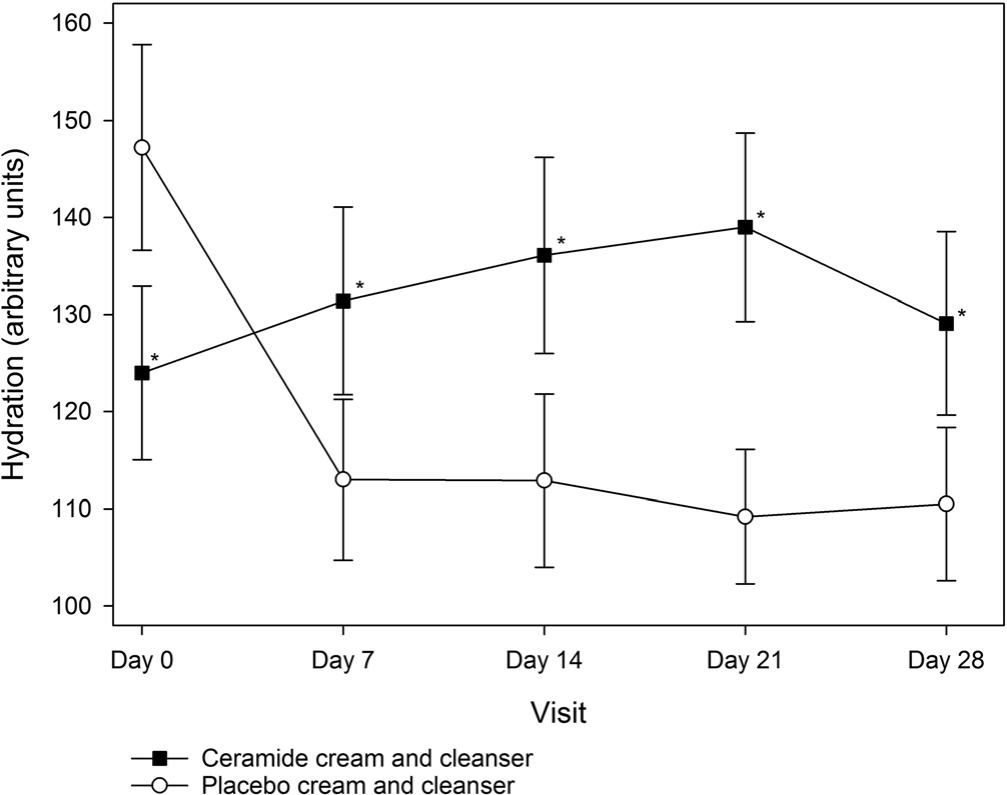
Skin hydration in the ceramide cream and cleanser and placebo cream and cleanser groups over the 28 day study period (ITT population). *P < .05 vs placebo

TEWL was similar in both groups at baseline (*P* > .05; Table 2). The active group had significantly greater improvements in TEWL at all‐time points compared to the placebo group which showed little to no improvement over the study period (Figure 3). This improvement was statistically significant at all‐time points (*P* < .05) except for day 21, where it approached significance (*P* = .0660). The difference in skin hydration between the active and placebo groups was significant at all‐time points (*P* < .05; Figure 4), with skin hydration consistently improving in the active group over time. Corroborating evidence from the mixed models analysis found both TEWL and skin hydration improved in the active group, while it either stayed the same or worsened in the placebo group (*P* = .0342 and *P* < .0001, respectively).

**FIGURE 5:**
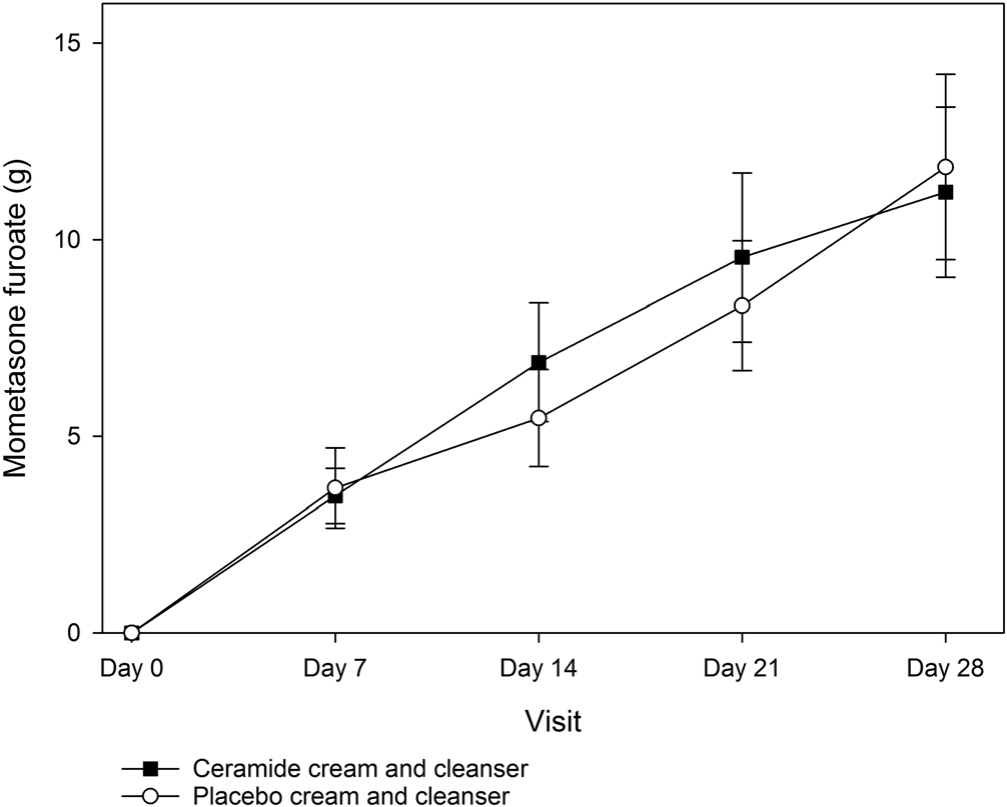
The amount of mometasone furoate used as rescue medication in the ceramide cream and cleanser and placebo cream and cleanser groups over the 28 day study period (ITT population)

There were no significant differences in the amount of mometasone furoate used as rescue medication by either group at any time point (all *P* > .05; Figure 5). Furthermore, there were no significant differences in the quantity of cream or cleanser used between both groups at any time point (all *P* > .05; data not shown).

**FIGURE 6:**
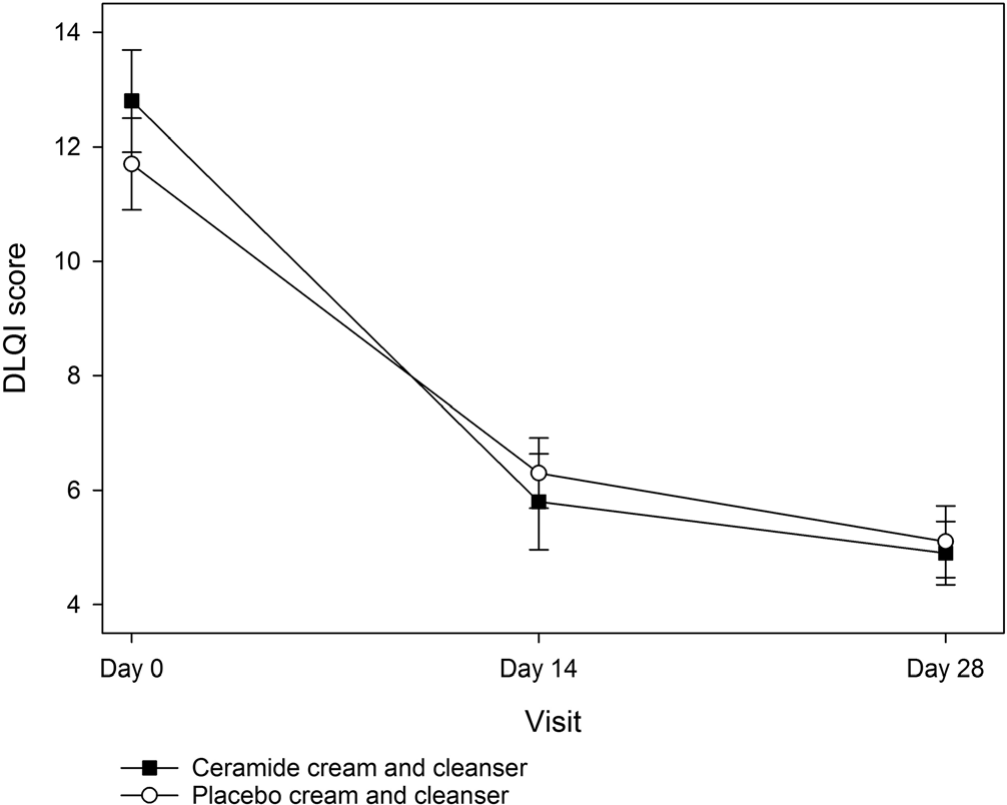
Dermatology Life Quality Index (DLQI) scores in the ceramide cream and cleanser and placebo cream and cleanser groups over the 28 day study period (ITT population)

Baseline DLQI scores were similar in both groups (*P* > .05; Table 2). DLQI scores improved significantly over time in both groups (*P* < .0001; Figure 6), with no significant difference observed between groups (*P* = .7804). However, analysis of the patient satisfaction survey by cumulative logistic regression (Table 3) found that several questions were answered more positively in the active compared to the placebo group, including relief of itch on day 14 (*P* = .0255), relief of dry skin at both day 14 (*P* < .0001) and day 28 (*P* = .0033) and effects on skin softness and smoothness at day 14 (*P* = .0001) and day 28 (*P* = .0573). The reduction of rash approached significance at day 14 (*P* = .0698). No differences were found between groups for the reduction of redness and inflammation, treatment pleasantness, maintenance of healthy skin, ease of use and overall satisfaction (all *P* > .05).

**TABLE 3:** Cumulative logistic regression analysis of patient satisfaction survey questions following the use of either ceramide cream and cleanser or placebo cream and cleanser at day 14 and 28

Category	Visit	Odds Ratio	SE	95% Confidence Interval	*P* value
Relief of itch	**Day 14**	**2.5466**	**1.0655**	**1.1215 to 5.7826**	**.0255**
	Day 28	0.8638	0.3461	0.3938 to 1.8944	.7147
Reduction of redness and inflammation	Day 14	1.5636	0.6136	0.7246 to 3.3740	.2547
	Day 28	0.7996	0.3327	0.3538 to 1.8072	.5909
Relief of dry skin	**Day 14**	**5.4131**	**2.2455**	**2.4007 to 12.2052**	**<.0001**
	**Day 28**	**3.5914**	**1.5648**	**1.5290 to 8.4360**	**.0033**
Reduction of rash	Day 14	2.0453	0.807	0.9438 to 4.4322	.0698
	Day 28	0.7584	0.3184	0.3331 to 1.7267	.5101
Product effect on skin softness and smoothness	**Day 14**	**5.0445**	**2.1159**	**2.2171 to 11.4775**	**.0001**
	**Day 28**	**2.1812**	**0.8948**	**0.9761 to 4.8741**	**.0573**
Product use pleasantness	Day 14	0.5892	0.2415	0.2638 to 1.3159	.1969
	Day 28	0.5137	0.2051	0.2349 to 1.1234	.0952
Product maintenance of healthy skin	Day 14	1.8382	0.7251	0.8484 to 3.9826	.1228
	Day 28	1.2273	0.5124	0.5415 to 2.7817	.6237
Product ease of use	Day 14	0.8607	0.3731	0.3681 to 2.0128	.7294
	Day 28	0.7131	0.3306	0.2874 to 1.7692	.4658
Overall satisfaction with product	Day 14	1.7307	0.6305	0.8475 to 3.5345	.1321
	Day 28	0.9928	0.4333	0.4220 to 2.3356	.9867

Abbreviation: ITT, intention‐to‐treat.

*Note*: Statistically significant parameters are in bold. (ITT population).

### 3.3 Safety assessment

There were a small number of AEs (22) experienced by 18 patients (18%), with 11 (11%) of those patients in the active group and 7 (7%) in the placebo group. Of these, 8 patients (8%) were found to have 10 AEs that were remotely, possibly or probably related to the study products in the active group compared to 4 patients (4%) and 5 AEs in the placebo group. The most common treatment‐related AEs reported were pain (stinging on application) in five patients (5%) in the active group only, itch in three patients (3%) in the active group and one (1%) in the placebo group, and dry skin in the placebo group only by two patients (2%). Two patients (2%) in the active group reported both pain and itch. The majority of treatment‐related AEs experienced by patients were classified as mild with five in the active group and five in the placebo group experienced by four patients (4%) in each group. Two patients (2%) experienced moderate severity AEs while two (2%) experienced severe AEs, all of which were in the active group; one patient experienced both pain and itch while the other three experienced pain only. No subjects withdrew from the study due to AEs and there were no serious AEs.

## 4 DISCUSSION

In this study, mean EASI score significantly improved by approximately 45% in patients with moderate eczema following use of a ceramide‐dominant physiological lipid‐based moisturizing cream and cleanser for 4 weeks. A similar outcome was also found for the placebo group and there was no difference in the use of mometasone furoate as rescue medication over time between groups. Strikingly though, use of the ceramide cream and cleanser resulted in significant improvements in barrier function compared to the placebo as measured by a decrease in TEWL and increased skin hydration. In addition, while differences in the DLQI scores between groups were not found, patient satisfaction was greater in the active compared to the placebo group for the relief of itch, relief of dry skin and the effects on skin smoothness and softness.

The positive effect of the ceramide cream and cleanser on restoring skin barrier function is most likely due to the presence of unique ingredients (Table 1) which have different mechanisms of action. Comprising a “triple moisturizing system”,^20^ ceramide cream and cleanser contain glycerin and sodium PCA as humectants to attract and hold water in the SC and epidermis, dimethicone and petrolatum as occludents to maintain the increased water content in the skin, and paraffinum liquidum, hexanediol and caprylyl glycol as emollients to smooth rough skin created by improperly desquamating corneocytes.^21^ The benefits obtained through the use of these traditional moisturizing ingredients are further enhanced by additional ingredients targeted to assist in correcting the epidermal barrier dysfunction.^13^

Ceramide cream and cleanser also contain ceramide EOP and ceramide NP, cholesterol and linoleic acid from safflower oil in a 3:1:1 M ratio. These ingredients must be delivered in the correct ratio to have a positive effect on the integrity of the skin barrier^22^ since application in the incorrect ratio has been shown to impede barrier repair.^23^ Ceramide EOP and ceramide NP were utilized as these ceramides have been demonstrated to be deficient in eczematous skin.^5^ Furthermore, topical delivery of ceramides has also been shown to relieve itch.^24^ In addition, the ceramide cream and cleanser also contain pyroglutamic acid (PCA), lactic acid and nicotinamide to promote and enhance the effects of ceramides. PCA, which is a filaggrin breakdown product and part of the skin's natural moisturizing factor (NMF), is present as sodium PCA, the form of PCA most used in topical preparations, which helps to restore the hydration of the SC.^25^ Lactic acid also forms part of the NMF, and together with nicotinamide have been shown to promote ceramide biosynthesis and thus further strengthen the skin barrier.^26^, ^27^

Similar outcomes to those observed in this study have been reported in the relatively few clinical studies examining the safety and efficacy of topical physiologic lipids in eczema. For example, adults with AD treated with mometasone furoate in combination with a ceramide and linoleic acid moisturizer for 8 weeks experienced accelerated reestablishment of the epidermal permeability barrier and amelioration of itch compared to treatment with mometasone furoate only.^28^ In another study, use of a ceramide‐dominant triple‐lipid barrier repair formulation for 28 days in children with moderate‐to‐severe AD resulted in reduced clinical disease severity and itch and improved sleep habits compared to treatment with fluticasone cream.^29^ In addition, other clinical studies have shown that moisturizers containing ceramides can be used to prolong the time between eczema flares,^30^, ^31^ and can also reduce the incidence of eczema developing in high‐risk infants with a family history of the condition.^32^, ^33^

Similar results have been observed in adults and children using moisturizers containing synthetic pseudoceramides^34^, ^35^, ^36^ or ceramide precursor lipids.^37^, ^38^ However, the nature of pseudoceramides and ceramide precursors may make them less efficacious in treating dry skin than ceramides.^20^, ^24^

This study demonstrates the importance of supporting the barrier function of eczematous skin and highlights the need for moisturizers and cleansers to be formulated specifically for eczema. A limitation of the study is that patients were not followed up after completion of the study to determine whether the skin barrier continued to improve. Furthermore, a change in EASI may have been observed if a longer study period was used. Similar studies of up to 8 weeks with a follow‐up period may be useful in both adults and children with moderate eczema.

## 5 CONCLUSION

This is the first study to show clinical evidence that a commercially available moisturizing cream and cleanser containing ceramides and other lipids in the appropriate physiological ratio, successfully and safely improves the signs and symptoms of moderate eczema in adults.

## CONFLICT OF INTEREST

Fabrizio Spada, Ian P. Harrison, Tanya M. Barnes, Kerryn A. Greive, Daisy Daniels and Joshua P. Townley are employed by Ego Pharmaceuticals, the sponsor of the study and manufacturer of the ceramide cream and cleanser.

## AUTHOR CONTRIBUTIONS

Fabrizio Spada and Ian P. Harrison were involved in clinical trial management, data interpretation and manuscript preparation. Tanya M. Barnes was involved in clinical trial design, data interpretation and manuscript preparation. Kerryn A. Greive was involved in clinical trial design and management. Daisy Daniels was involved in clinical trial management and Joshua P. Townley was involved in data interpretation. Niyaz Mostafa, Andrew T. Fong and Philip L. Tong were involved in patient recruitment, assessment of patient outcomes, data collection and manuscript preparation. Stephen Shumack was involved in assessment of patient outcomes, data collection, manuscript preparation and final approval.

## Data Availability

The data that support the findings of this study are available from the corresponding author upon reasonable request.
